# Lycopene Mitigates Malaria‐Induced Reactive Oxygen and Nitrogen Species and Oxidative Damage in Mice Brain and Lungs

**DOI:** 10.1111/pim.70019

**Published:** 2025-08-23

**Authors:** Everton Luiz Pompeu Varela, Antônio Rafael Quadros Gomes, Aline da Silva Barbosa dos Santos, Mariana dos Santos Guimarães, Eliete Pereira de Carvalho, Oberdan Oliveira Ferreira, Mozaniel Santana de Oliveira, Marcello Iriti, Eloisa Helena de Aguiar Andrade, Sandro Percário

**Affiliations:** ^1^ Oxidative Stress Research Laboratory, Institute of Biological Sciences Federal University of Pará Belém Pará Brazil; ^2^ Postgraduate Program in Biodiversity and Biotechnology – BIONORTE Network Federal University of Pará Belém Pará Brazil; ^3^ Adolpho Ducke Laboratory, Botany Coordination Museu Paraense Emílio Goeldi Belém Pará Brazil; ^4^ Laboratory of Pharmacology of Inflammation and Behavior, Postgraduate Program in Pharmaceutical Sciences, Institute of Health Sciences Federal University of Pará Belém Brazil; ^5^ Department of Biomedical, Surgical and Dental Sciences University of Milan Milan Italy; ^6^ National Interuniversity Consortium of Materials Science and Technology (INSTM) Firenze Italy

**Keywords:** antioxidant, lycopene, malaria, N‐acetylcysteine, oxidative stress

## Abstract

The severity of malaria is associated with low antioxidant availability and elevated free radical production, which induces oxidative damage in cerebral and pulmonary microcirculation. This can be mitigated by dietary antioxidants. We investigated the protective effects of lycopene (LYC) against oxidative changes induced by *Plasmodium berghei* (Pb). Mice were infected by intraperitoneal injection of 10^6^ parasitized red blood cells and treated orally with LYC (3.11 mg/kg bw/day) or N‐acetylcysteine (NAC, 62 mg/kg bw/day). Evaluations were conducted at 1‐, 4‐, 8‐ and 12‐days post‐infection. We measured thiobarbituric acid reactive substances (TBARS), antioxidant capacity by ABTS (AC‐ABTS) and DPPH (AC‐DPPH) inhibition, uric acid (UA) and nitric oxide (NO) in brain and lung tissues. Infection led to elevated TBARS, AC‐ABTS, AC‐DPPH, UA and NO, resulting in animal mortality. LYC significantly attenuated the infection‐induced increases in TBARS, UA and NO levels compared to Pb (*p* < 0.0001) and NAC + Pb groups (*p* < 0.0001) normalising them to Sham levels. These findings highlight LYC's therapeutic potential against malaria‐related oxidative stress.

## 1 Introduction

Free radicals are intermediate molecular species characterised by the presence of unpaired electrons. The generation of these reactive molecules is an unavoidable consequence of physiological metabolism, as many arise from the physicochemical oxidation of molecular oxygen (O_2_) and nitrogen (N_2_), resulting in the formation of reactive oxygen and nitrogen species (RONS) [1]. RONS are highly oxidising intermediates continuously produced in biological systems through various biochemical pathways, including nitric oxide (NO) synthesis, mitochondrial electron transport chain activity and metal‐catalysed redox reactions [2].

The primary RONS generated in the human body include superoxide anion (O_2_˙^−^), hydrogen peroxide (H_2_O_2_), hydroxyl radical (OH˙), singlet oxygen (^1^O_2_), NO, nitrogen dioxide (NO_2_) and peroxynitrite (ONOO^−^) [3]. Additional biologically relevant reactive species include lipid hydroperoxides (ROOH), lipid peroxyl radical (ROO˙) and lipid alkoxyl radical (RO˙). These species are highly unstable and reactive due to their unpaired electrons, which confer a short half‐life, often measured in milliseconds, but sufficient to engage in redox interactions with neighbouring biomolecules, leading to molecular alterations and tissue damage [4].

To counteract the harmful effects of RONS, the human body activates an array of mobilisable antioxidant defence mechanisms that function to prevent, neutralise, or mitigate oxidative reactions [5, 6]. Key antioxidant enzymes include superoxide dismutase (SOD), catalase (CAT) and glutathione peroxidase (GSH‐Px), which are responsible for converting O_2_˙^−^ and decomposing H_2_O_2_ and ROOH into less reactive molecules such as water, alcohol and oxygen [7].

However, when antioxidant defences are insufficient to neutralise excessive RONS production, the organism experiences oxidative stress. Oxidative stress is thus defined as an imbalance between RONS generation and antioxidant capacity in favour of RONS accumulation [8]. While oxidative stress plays a physiological role in immune defence, mediating cytotoxic activity against pathogens, it can also contribute to pathogenesis by disrupting redox signalling and inducing damage to lipids, proteins and nucleic acids. This redox imbalance is implicated in the development of chronic, degenerative, neurodegenerative, metabolic and infectious diseases [4, 7].

**FIGURE 1:**
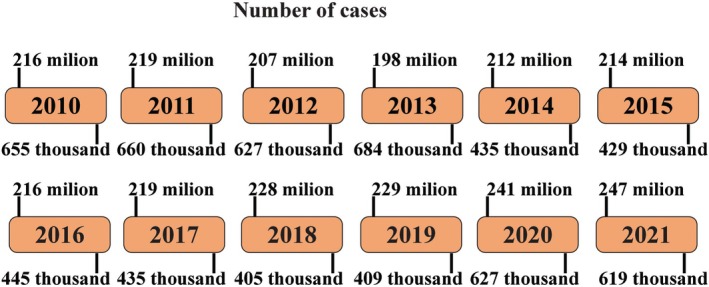
Timeline of the number of cases (above) and number of deaths (below) of malaria in the world.

Within this framework, increasing attention has been directed toward the role of oxidative stress and antioxidant defences in the pathophysiology of malaria [9]. Malaria remains a global health concern, responsible for more than 200 million clinical episodes and approximately 500 000 deaths annually, primarily affecting populations in low‐income regions (Figure 1) [10]. Among the various species of *Plasmodium, P*. *falciparum* and *P. vivax* are the most significant threats to human health.

RONS are associated with the various pathological manifestations in malaria, ranging from mild symptoms such as fever and anaemia to severe complications including cerebral malaria, respiratory distress, metabolic acidosis, hepatic and renal failure and profound anaemia [11, 12, 13]. According to Suzuki et al. [14], these outcomes are, in part, due to the depletion of mobilisable antioxidants consumed during oxidative stress. Additionally, ischaemia–reperfusion syndrome (IRS), the host immune response and parasite metabolism further exacerbate RONS accumulation [5, 6, 12].

During infection, *Plasmodium* parasites invade red blood cells and degrade haemoglobin to obtain amino acids. This process releases free heme (Fe^2+^), which participates in Fenton and Haber‐Weiss reactions that generate O_2_˙^−^, H_2_O_2_ and OH˙, contributing to oxidative damage in lipids, proteins and DNA [15, 16, 17]. Moreover, RONS can activate toll‐like receptor‐4 (TLR4), promoting an immune response. According to Ty et al. [18], RONS initiate the respiratory burst in neutrophils and macrophages, enhancing parasite clearance, primarily through NO production.

NO is a gaseous free radical synthesised from L‐arginine by nitric oxide synthase (NOS). While constitutive NOS isoforms (eNOS in endothelial cells and nNOS in neurons) produce NO at steady levels, inducible NOS (iNOS) in macrophages generates large quantities of NO in response to infection [19, 20, 21]. Despite its protective role, excessive NO reacts with O_2_˙^−^ to form ONOO^−^, a highly oxidising molecule that promotes endothelial apoptosis, cytoadherence and microvascular obstruction [22, 23]. Additionally, NO can bind to haemoglobin, impairing oxygen delivery and exacerbating tissue hypoxia and anaemia [24].

Thus, the excessive oxidative burden, driven by RONS and hemolytic processes, contributes to severe outcomes such as vascular occlusion, acidosis and multi‐organ damage [5, 11, 12, 19]. However, this damage can be mitigated by exogenous antioxidants found in food. These include vitamins E and C, phenolic compounds (e.g., flavonoids, resveratrol), carotenoids (e.g., β‐carotene, lycopene [LYC]), and pharmaceutical agents such as N‐acetylcysteine (NAC) [5, 6].

Among these, LYC, a naturally occurring, lipophilic carotenoid found in tomatoes, watermelon and carrots, has attracted growing attention due to its potent antioxidant, anti‐inflammatory, anticancer, neuroprotective and cardioprotective properties [25, 26, 27, 28, 29, 30, 31, 32, 33, 34, 35]. LYC scavenges a wide range of radicals (e.g., O_2_˙^−^, OH˙, NO, ONOO^−^) via electron and hydrogen atom transfer, with antioxidant capacity reportedly up to 100 times greater than that of vitamin E, due to its conjugated double‐bond system [25, 36, 37].

Several studies have shown that LYC supplementation reduces oxidative stress markers such as malondialdehyde (MDA), NO and inflammatory cytokines [38, 39, 40]. For instance, Pan et al. [41] demonstrated that LYC prevents cyclosporine‐induced intestinal injury by reducing oxidative stress and inflammation in mice. These findings highlight the LYC's ability to combat RONS‐mediated damage in malaria [6].

Therefore, this study aimed to investigate whether LYC, a potent dietary antioxidant, could mitigate oxidative stress induced by *P. berghei* infection in a murine model. Given its strong RONS‐scavenging properties and tissue bioaccumulation, we hypothesised that LYC treatment would significantly reduce oxidative stress biomarkers in brain and lung tissues. Furthermore, by comparing LYC to NAC, a widely studied antioxidant, we aimed to evaluate its relative efficacy in modulating biochemical and oxidative parameters during experimental malaria. These findings may support the use of LYC as an adjuvant therapeutic strategy against malaria‐induced oxidative damage.

## 2 Materials and Methods

### 2.1 Subheading

A total of 231 adult male BALB/c mice, 7–10 weeks of age, weighing between 25 and 40 g (Vivarium of the Evandro Chagas Institute, Ananindeua, Pará‐Brazil) were used. The animals were housed in the Experimentation Vivarium of the Oxidative Stress Research Laboratory (LAPEO) of the Institute of Biological Sciences (ICB) of the Federal University of Pará (UFPA), at an ambient temperature of 24°C ± 2°C, a 12‐h light/dark cycle (lights from 7 a.m. to 7 p.m.), and *ad libitum* access to food and water. Before any experimental procedure was carried out, animals were allowed to acclimatise to laboratory conditions for 15 days.

All experimental procedures were performed at LAPEO. The animals were handled and cared for in accordance with the ethical standards of animal experimentation indicated by the Brazilian Society of Laboratory Animal Science. The project was approved by the Ethics Committee on the Use of Animals of UFPA (CEUA/UFPA) (approval no. 3235130919).

### 2.2 Protocol for the Preparation andAdministration of Lycopene and N‐Acetylcysteine

The protocol for preparation and administration of LYC was chosen based on a dose–response study on the effects of LYC supplementation on biomarkers of oxidative stress [42], and the dose was calculated by allometric extrapolation [43].

The NAC preparation and administration protocol was chosen based on a randomised, double‐blind, placebo‐controlled study of chronic obstructive pulmonary disease [44]; the dose was calculated by allometric extrapolation [43].

Mice in the LYC + Pb and NAC + Pb groups were pretreated with a dose of 3.11 mg/kg bw/day of LYC or 62 mg/kg bw/day of NAC by gavage, respectively, 24 h prior to infection. Treatments then continued daily until the day before the animals were euthanised. Mice of the Sham group received only vehicle (water) orally.

### 2.3 *Plasmodium berghei* ANKA *‐*Infection Protocol

The mice of the groups PB, LYC + Pb and NAC + Pb were performed by intraperitoneal injection (i.p.) of 10^6^ *P. berghei* ANKA‐parasitized red blood cells (pRBC). Conversely, the Sham group animals received 10^6^ of non‐parasitised red blood cells.

### 2.4 Protocol for Subdivision of the Experimental Groups

**FIGURE 2:**
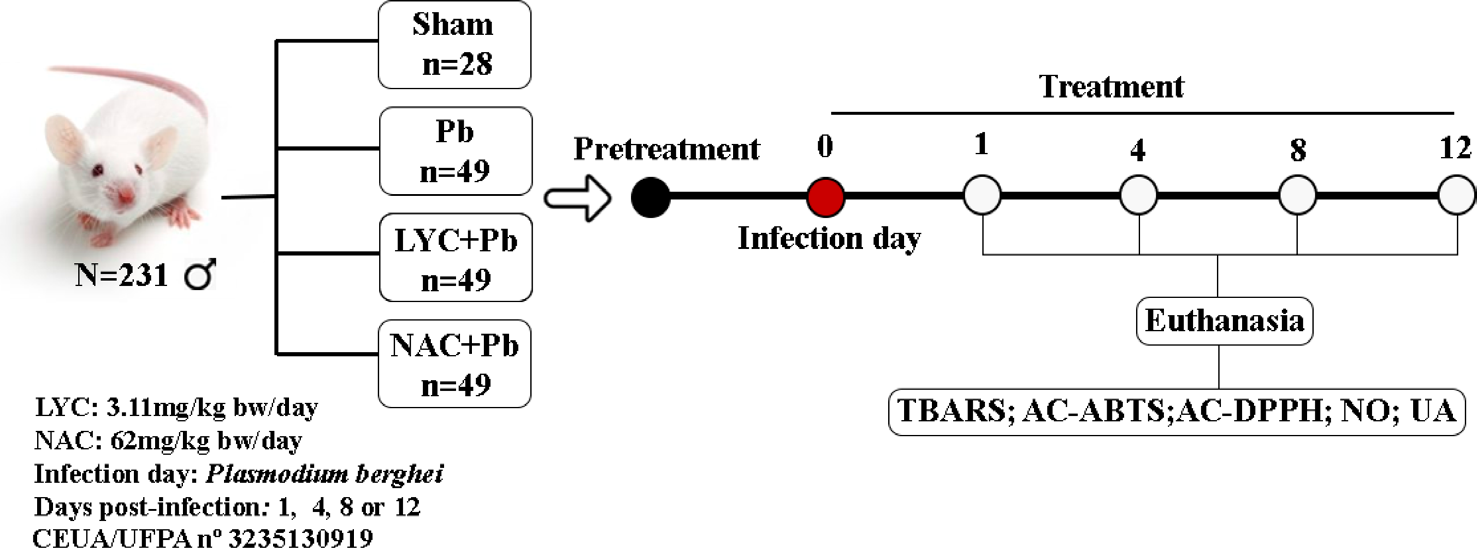
Schematic representation of the experimental protocol. BALB/c mice were pretreated with LYC or NAC prior to inoculation with 10^6^ pRBC, and treatment continued daily until the day before euthanasia, for 1, 4, 8, or 12 consecutive days. After euthanasia, brain and lungs were collected for biochemical analyses: Antioxidant Capacity by inhibition ABTS (AC‐ABTS) and DPPH (AC‐DPPH) radicals; NO, nitric oxide; TBARS, thiobarbituric acid reactive substances; UA, uric acid.

In an experiment of 1, 4, 8, or 12 days of consecutive follow‐up, 231 male mice (BALB/c) were randomly assigned to one of four groups (Figure 2), including **Sham** (*n* = 28): mice received the vehicle (water; orally) and non‐parasitised red blood cells (i.p.); **Pb** (*n* = 49): pRBC‐infected mice (i.p.); **LYC + Pb** (*n* = 49): LYC‐treated mice (orally) and pRBC‐infected mice (i.p.); **NAC + Pb** (*n* = 49): NAC‐treated mice (orally) and pRBC‐infected mice (i.p.).

The subgroups 1‐ and 4‐day comprised 7 animals each. The 8‐ and 12‐day subgroups comprised 15 and 20 animals, respectively, due to the higher mortality expected for these subgroups.

### 2.5 Euthanasia Protocol and Sample Preparation

At the end of each study period, each animal was anaesthetised (i.p.) using a combination of 0.5 mL of 10% ketamine hydrochloride (9 mg/kg) + 0.25 mL of 2% xylazine hydrochloride (10 mg/kg) + 4.25 mL of water for injection. After confirmation of unconsciousness and loss of corneal reflex, animals were euthanised by exsanguination through intracardiac puncture.

Subsequently, both lungs and the brain of each animal were extracted. The organs were weighed and placed in phosphate saline‐buffer solution (PBS) in a ratio of 1:10 (m:v). Subsequently, the ultrasonic disruption of tissues was performed to obtain a homogenate. After homogenisation, the material was centrifuged (80‐2B analog centrifuge; Daiki; Rio de Janeiro; Brazil) at 2500 rpm for 10 min; the supernatant was collected, stored in an Eppendorf microtube, and frozen at −20°C until assayed.

### 2.6 Biochemical Measurements Protocol

#### 2.6.1 Thiobarbituric Acid Reactive Substances

The method was carried out according to the fundamentals proposed by Kohn and Liversedge [45], with the chemical conditions of the reaction adjusted according to the protocol described by Percário et al. [46]. This method evaluates lipid peroxidation and has been used as an indicator of oxidative stress. The test is based on the reaction of thiobarbituric acid (4,6‐Dihydroxypyrimidine‐2‐thiol, TBA; Sigma‐Aldrich; T5500; São Paulo/SP) with by‐products of lipid peroxidation (e.g., MDA), at acidic pH (2.5) and high temperature (94°C), forming chromogens with absorbance at 535 nm.

Initially, 0.5 mL of the sample or standard was mixed with 1 mL of the TBA solution (10 mM). Then, this solution was placed in a water bath at 94°C for 60 min. Subsequently, 4 mL of n‐butyl alcohol was added; the solution was stirred in a vortex‐type agitator, then centrifuged (80‐2B analog centrifuge; Daiki; Rio de Janeiro; Brazil) at 3000 rpm for 10 min. After that, 3 mL of the supernatant was transferred to a cuvette, and absorbance was read at 535 nm using a spectrophotometer (Spectrophotometer 800XI; Femto; São Paulo/SP).

A standard curve (1,1,3,3, tetrahydroxypropane; standard MDA; 20 μM; Sigma‐Aldrich Chemical; 108383; São Paulo/SP) was performed in triplicate and, from the values obtained, the equation of the line (*y* = 0.1419*x* − 0.0037) was calculated, where y represents the absorbance value and *x* the concentration value, obtaining *R* ^2^ = 0.9999. From the equation of the line, the concentration of TBARS of the samples was determined.

#### 2.6.2 Antioxidant Capacity by Inhibition ABTS Radical

This test was carried out based on a methodology proposed by Miller et al. [47], with reaction conditions modified by Re et al. [48]. The method is based on the ability of substances to eliminate the radical cation 2,2′‐azino‐bis (3‐ethylbenzothiazoline‐6‐sulfonic acid) diammonium salt (ABTS˙^+^), a blue‐green chromophore with maximum absorption at 734 nm, resulting in the formation of a stable product, ABTS, which is colourless.

Initially, the ABTS˙^+^ solution (2.45 mM) was prepared from the reaction between ABTS (7 mM; Sigma‐Aldrich; A1888; São Paulo/SP) and potassium persulfate (140 mM; K_2_O_8_S_2_; Sigma‐Aldrich; 216,224; São Paulo/SP). Then, the initial absorbance reading (T0) of the ABTS˙^+^ solution was carried out at 734 nm in an 800XI spectrophotometer (Femto; São Paulo/SP). Then, 30 μL of sample or standard was added to the solution and, after an incubation period of 5 min, the final reading (T5) was performed by measuring the absorbance again.

A standard curve (6‐hydroxy‐2,5,7,8‐tetramethylcromono‐2‐carboxylic acid; Trolox; 2.5 mM; Sigma‐Aldrich; 23881‐3; São Paulo/SP) was performed in triplicate and, from the absorbance values found, the equation of the line (*y* = 0.4324*x* + 0.0049) was obtained, where *y* represents the absorbance value and *x* the concentration value, obtaining *R* ^2^ = 0.9997. From the equation of the line, the AC‐ABTS of the samples was determined.

#### 2.6.3 Antioxidant Capacity by Inhibition DPPH Radical

The test was performed according to an adapted method proposed by Blois [49] This assay evaluates the total antioxidant capacity of synthetic or natural substances to eliminate the DPPH˙ radical (Sigma‐Aldrich; D9132; São Paulo/SP), a violet chromophore with absorption at 517 nm, resulting in the formation of the hydrogenated product DPPH, which is yellow or colourless.

First, the DPPH˙ solution (0.1 mM) was prepared from the reaction between DPPH (394.32 g/mol; Sigma‐Aldrich; A1888; São Paulo/SP) and ethyl alcohol (P.A.; C_2_H_6_O; Sigma‐Aldrich; 216224; São Paulo/SP). Subsequently, the absorbance of the DPPH˙ solution at 517 nm was read in an 800XI spectrophotometer (Femto; São Paulo/SP). Then, 50 μL of the sample or standard was mixed in 950 μL of the DPPH˙ solution and placed in a water bath at 30°C for 30 min. After this period, the second reading was performed.

A standard curve (Trolox; 2.5 mM) was performed in triplicate and, from the absorbance values found, the equation of the line was obtained: *y* = 0.2041*x* − 0.0031, where *y* represents the absorbance value and *x* the concentration value, obtaining *R* ^2^ = 0.9973. From the equation of the line, the AC‐DPPH was determined in the samples.

#### 2.6.4 Nitric Oxide

The NO concentration was determined indirectly by the detection of nitrate (NO_3_−) or nitrite (NO_2_^−^) in the samples, using an NO colorimetric assay kit (Elabscience, Catalogue No: E‐BC‐K035‐M). NO is readily oxidised to form nitrite in vivo or in aqueous solution, which can react with the chromogenic reagent nitrate, forming a pale red compound. The concentration of the compound is linearly related to the concentration of NO in the sample.

Initially, 100 μL of sample or standard was mixed with 200 μL of reagent 1 (sulfate solution) and 100 μL of reagent 2 (alkaline reagent). After being allowed to rest for 15 min, the solution was centrifuged at 3000 rpm for 10 min. Then, 160 μL of the supernatant was transferred to a microplate, where 80 μL of the chromogenic reagent was added. After 15 min of incubation at room temperature, absorbance was read at 550 nm using a spectrophotometer. A standard curve (sodium nitrite; 100 μM) was performed in triplicate; and, from the values found, the equation of the line was obtained (*y* = 0.0022*x* − 0.0005), where y represents the absorbance value and *x*, the concentration value, obtaining *R* ^2^ = 0.9987. From the equation of the line, the concentration of NO in the samples was determined.

#### 2.6.5 Uric Acid

The procedure was performed using the Liquiform Uric Acid Kit (Labtest). The technique consists of the oxidation of uric acid by uricase producing allantoin and H_2_O_2_. H_2_O_2_ in the presence of peroxidase reacts with 3,5‐dichloro‐2‐hydroxybenzene sulfonate acid (DHBS) and 4‐aminoantipyrine to form the antipyrylquinonymine chromogen. The intensity of the red colour formed is directly proportional to the concentration of uric acid in the sample.

To perform the assay, 0.02 mL of the sample or standard was mixed in 1 mL of UA working reagent (4‐aminoantipyrine, peroxidase, sodium azide, DHBS and uricase). The solution was then incubated in a water bath at 37°C for 5 min. Then, absorbance was read at 505 nm using an 800XI spectrophotometer (Femto; São Paulo/SP).

A standard curve (UA; 20 mg/dL) was performed in triplicate and, from the values found, the equation of the line was obtained (*y* = 0.0166*x* + 0.0012), where *y* represents the absorbance value and *x* the concentration value, obtaining *R* ^2^ = 0.9986. From the equation of the line, the concentration of UA in the samples was determined.

### 2.7 Statistical Analysis

For each parameter examined, the analysis of possible outliers was performed by calculating the interquartile range, in which the difference between the third quartile (Q3) and the first quartile (Q1), termed dj, was determined. Any value lower than Q1‐3/2dj or greater than Q3 + 3/2dj was considered to be an outlier and was not taken into consideration in the statistical calculations. After the analysis of the discrepant points, normality was assessed using the Levene test. For homoscedastic distribution, the analysis of variance (ANOVA) test was applied, and for heteroscedastic dispersion, the Kruskal–Wallis test was applied. Significant differences were compared between the groups using Tukey's *post hoc* test.

In the intragroup temporal progression analysis, the unpaired Student's *t*‐test was performed. To verify the possible correlation between parameters, Pearson's correlation test was performed, considering the paired values of two parameters obtained for the same animal, and the calculations were performed using the data obtained from all animals simultaneously, according to the group to which they belonged. For the pairs of values in which a linear relationship was suspected to exist, regression analysis was performed, using all animals in both groups simultaneously and each group individually. In all tests, a significance level of 5% (*p* ≤ 0.05) was considered.

## 3 Results

**FIGURE 3:**
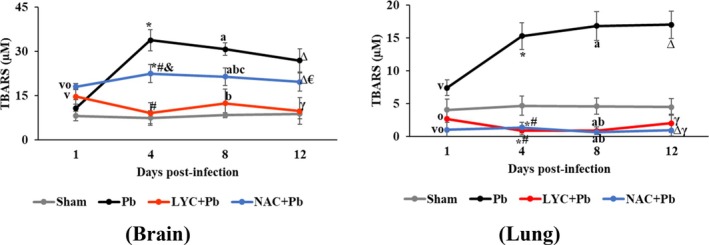
Levels of TBARS in the brain and lungs of pRBC‐infected mice treated with LYC or NAC. Data are expressed as means ± standard deviation. 1 day: ^V^ *p* ≤ 0.02 versus Sham; ^o^ *p* ≤ 0.005 versus Pb. 4 days: *p ≤ 0.001 versus Sham; ^#^ *p* < 0.0001 versus Pb; ^&^ *p* < 0.0001 versus LYC + Pb. 8 days: ^A^p ≤ 0.0002 versus Sham; ^b^ *p* < 0.0001 versus Pb; ^c^ *p* < 0.0001 versus LYC + Pb. 12 days: ^∆^ *p* < 0.0001 versus Sham; ^γ^ *p* < 0.0001 versus Pb; ^€^ *p* < 0.0001 versus LYC + Pb.

Our results demonstrated that *P. berghei* infection significantly increased TBARS levels compared to the Sham group in brain and lung tissues on Days 4, 8 and 12 post‐infection (*p* < 0.0001; Figure 3). Both the LYC + Pb and NAC + Pb groups exhibited significantly lower TBARS levels than the Pb group in both tissues (*p* < 0.0001). In addition, LYC treatment appeared to normalise TBARS levels, bringing them close to those observed in the brain and lung tissues of the Sham group.

**FIGURE 4:**
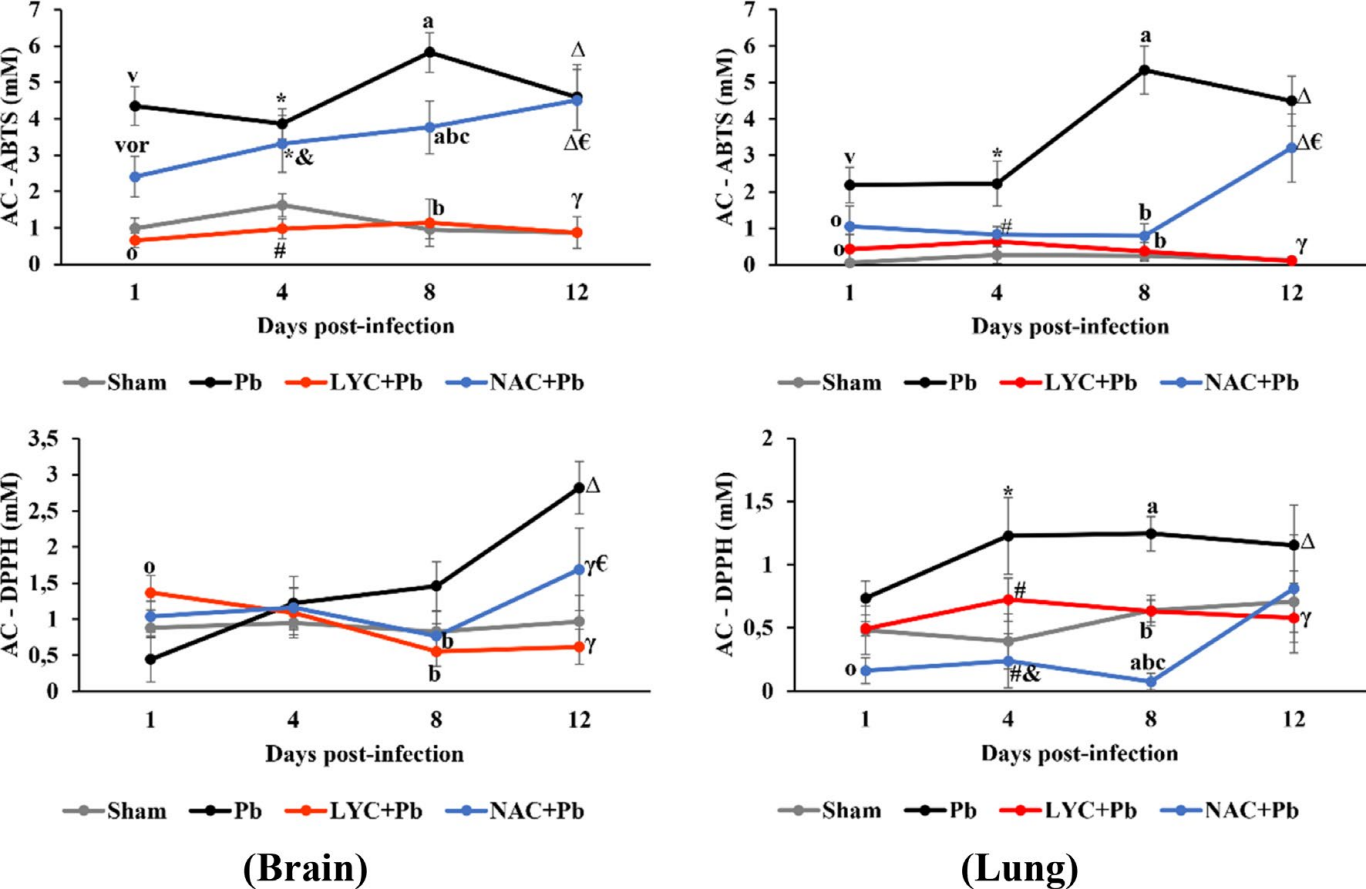
Antioxidant capacity by inhibition ABTS and DPPH radicals in the brain and lungs of pRBC‐infected mice treated with LYC or NAC. Data are expressed as means ± standard deviation. 1 day: ^V^ *p* < 0.0001 versus Sham; ^o^ *p* ≤ 0.005 versus Pb; ^r^ *p* < 0.0001 versus LYC + Pb. 4 days: *p ≤ 0.0001 versus Sham; ^#^ *p* ≤ 0.01 versus Pb; ^&^ *p* ≤ 0.02 versus LYC + Pb. 8 days: ^A^ *p* < 0.0001 versus Sham; ^b^ *p* < 0.0001 versus Pb; ^c^ *p* < 0.0001 versus LYC + Pb. 12 days: ^∆^ *p* ≤ 0.005 versus Sham; ^γ^ *p* ≤ 0.001 versus Pb; ^€^ *p* < 0.0001 versus LYC + Pb.

We observed that *P. berghei* infection led to a significant increase in AC‐ABTS and AC‐DPPH levels in the Pb group compared to the Sham group in both brain and lung tissues (*p* < 0.0001; Figure 4). In the LYC group, a significant decrease in AC‐ABTS and AC‐DPPH levels was observed compared to the Pb group in both tissues (*p* < 0.0001), showing a pattern similar to that of the Sham group. However, treatment with NAC resulted in a significant increase in AC‐ABTS levels in brain tissue compared to the Sham and LYC groups (*p* < 0.0001).

**FIGURE 5:**
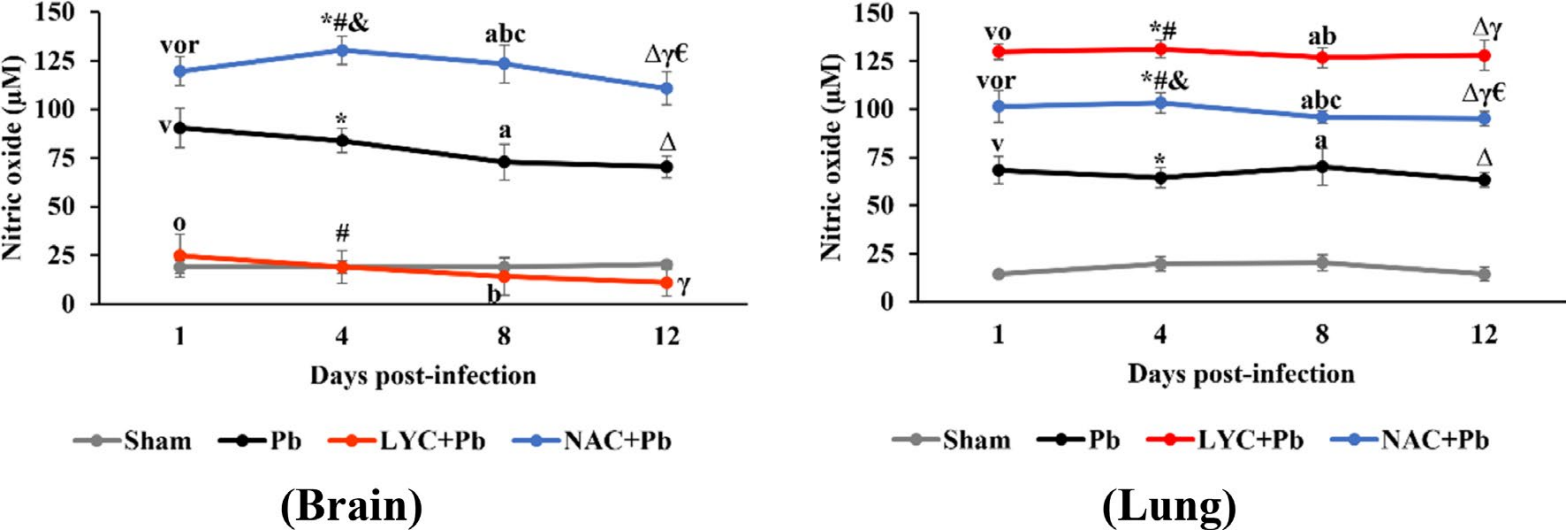
Nitric oxide concentration in the brain and lungs of pRBC‐infected mice treated with LYC or NAC. Data are expressed as means ± standard deviation. 1 day: ^V^ *p* < 0.0001 versus Sham; ^o^ *p* < 0.0001 versus Pb; ^r^ *p* < 0.0001 versus LYC + Pb. 4 days: *p < 0.0001 versus Sham; ^#^ *p* < 0.0001 versus Pb; ^&^ *p* < 0.0001 versus LYC + Pb. 8 days: ^A^ *p* < 0.0001 versus Sham; ^b^p < 0.0001 versus Pb; ^c^ *p* < 0.0001 versus LYC + Pb. 12 days: ^∆^ *p* < 0.0001 versus Sham; ^γ^ *p* < 0.0001 versus Pb; ^€^ *p* < 0.0001 versus LYC + Pb.

*P. berghei* induced a significant increase in NO levels in the Pb group compared to the Sham group in both brain and lung tissues throughout the study period (*p* < 0.0001; Figure 5). Following treatment with LYC, a significant reduction in NO level was observed in brain tissue compared to the Pb and NAC + Pb groups (*p* < 0.0001). Moreover, LYC treatment normalised NO level in brain tissue. Conversely, in lung tissue, LYC treatment induced a significant increase in NO levels compared to all other groups (*p* < 0.0001).

**FIGURE 6:**
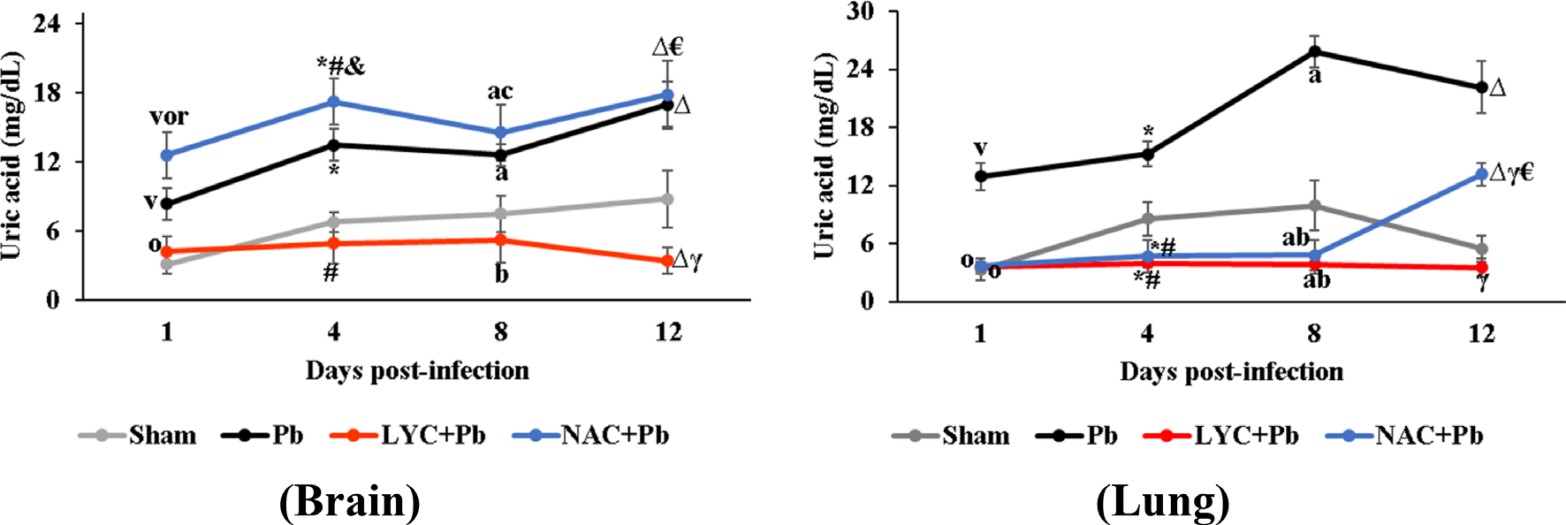
Uric acid concentration in brain and lungs of pRBC‐infected mice treated with LYC or NAC. Data are expressed as means ± standard deviation. 1 day: ^V^ *p* ≤ 0.0001 vs. Sham; ^o^ *p* ≤ 0.005 versus Pb; ^r^ *p* < 0.0001 versus LYC + Pb. 4 days: *p ≤ 0.0007 versus Sham; ^#^ *p* ≤ 0.02 versus Pb; ^&^ *p* < 0.0001 versus LYC + Pb. 8 days: ^A^ *p* < 0.0001 versus Sham; ^b^ *p* < 0.0001 versus Pb; ^c^ *p* < 0.0001 versus LYC + Pb. 12 days: ^∆^ *p* < 0.0001 versus Sham; ^γ^ *p* < 0.0001 versus Pb; ^€^ *p* < 0.0001 versus LYC + Pb.

*P. berghei* induced a significant increase in UA levels in the Pb group compared to the Sham group in both tissues (*p* < 0.0001; Figure 6). In contrast, LYC supplementation led to a significant reduction in UA levels compared to the Pb group in both tissues, and to the NAC + Pb group in brain tissue (*p* < 0.0001). LYC treatment normalised UA levels in both tissues, a pattern also observed in the lung tissue of the NAC‐treated group up to the eighth day of treatment.

## 4 Discussion

Malaria causes more than 500 000 deaths annually, most of which are due to brain and/or lung complications induced by *P. falciparum* infection. To better understand the pathophysiology of malaria, experimental infection in a mouse model using *P. berghei* has been widely employed, given its ability to induce oxidative biochemical changes, such as lipid peroxidation and decreased antioxidant capacity, in vital organs like the lung and brain, showing several similarities to humans disease [50, 51].

To investigate whether substances with antioxidant potential could mitigate oxidative biochemical changes during malarial infection, BALB/c mice were infected with *P*. *berghei* and treated with either LYC or NAC.

NAC was selected as a reference compound because it is widely used for the treatment and/or prevention of several respiratory diseases and has been shown to reduce oxidative stress in conditions including human immunodeficiency virus (HIV) infections, influenza A/H1N1 virus and malaria [52, 53, 54, 55]. LYC, an orally administered antioxidant, was tested for its antioxidant activity in *P. berghei*‐infected mice, and its effects were compared to those of NAC.

LYC is found in foods such as tomatoes, watermelon and guava, and possesses antioxidant potential approximately twice that of β‐carotene [56]. It is a lipophilic carotenoid absorbed in the small intestine via passive diffusion and protein‐mediated transport, particularly through the scavenger receptor class B type 1 (SR‐B1) [57]. After incorporation into chylomicrons, LYC enters systemic circulation and is transported by lipoproteins to various organs.

Notably, LYC accumulates in lipid‐rich tissues, including the liver, lungs and brain [58, 59]. Studies have confirmed its ability to cross the blood–brain barrier, making it a promising candidate for neuroprotective applications [60]. Its antioxidant activity is primarily attributed to its extended conjugated double bond system, enabling efficient quenching of singlet oxygen and neutralisation of RONS [61]. Its antioxidant property contributes to maintaining health and reducing the risk of oxidative stress‐related diseases, including cancer and malaria [38, 62].

In the present study, our data demonstrated that both LYC and NAC improved several biochemical markers associated with oxidative stress. However, LYC exerted a more pronounced effect than NAC. LYC treatment significantly reduced UA, TBARS and NO levels in *P. berghei*‐infected mice, lowering them to concentrations not only those observed in untreated infected animals, but also below those treated with NAC, approaching values observed in the uninfected (Sham) group.

The superior efficacy of LYC compared to NAC may be attributed to differences in their physicochemical properties and mechanisms of action. NAC is a hydrophilic thiol compound that replenishes intracellular glutathione (GSH) and acts primarily in the aqueous compartments of the cell. In contrast, LYC is highly lipophilic and preferentially localises within lipid‐rich membranes [63]. This lipophilicity allows LYC to directly protect cellular membranes from peroxidative damage caused by RONS, a mechanism less effectively addressed by NAC [64]. Furthermore, LYC's ability to neutralise a broader range of radicals through both electron and hydrogen atom transfer may explain its more consistent normalisation of oxidative stress biomarkers in both brain and lung tissues [65].

Although there is still no consensus on the precise mechanism underlying malaria severity, studies indicate that both the host and the parasite are subject to oxidative stress. This condition is driven by increased levels of circulating RONS, generated during the inflammatory response by activated immune cells such as monocytes and neutrophils, and primarily due to the degradation of haemoglobin by the parasite itself [6, 12, 66, 67, 68].

During infection, the parasite rapidly proliferates and consumes haemoglobin from the host erythrocytes, breaking it down into amino acids for its own nutrition. The process also releases Fe^2+^ into the cytosol, which undergoes Fenton and Haber–Weiss reactions in the presence of O_2_, leading to the generation of RONS such as O_2_˙^−^, H_2_O_2_ and OH^•^. These RONS promote lipid peroxidation, damage endothelial cells in microvessels, and harm vital tissues [15, 17].

In our study, we demonstrated that experimental malaria induced by *P*. *berghei* infection had a detrimental impact on oxidative biochemical parameters in mice, resulting in significantly increased levels of TBARS, AC‐DPPH, AC‐ABTS, NO and UA. This finding was expected, as Baptista et al. [69] reported that 60% of mice infected with *P. berghei* succumb to diseases between Days 6 and 8 post‐infection, even with moderate parasitemia ranging between 6% and 11%.

To assess oxidative biochemical alterations caused by RONS, we measured TBARS levels in lung and brain tissues of infected BALB/c mice. TBARS mainly include reactive α and β unsaturated aldehydes, such as MDA, 4‐hydroxy‐2‐nonenal, 2‐propenal (acrolein) and isoprostanes, which are secondary products of the decomposition of polyunsaturated fatty acid hydroperoxides. TBARS serve as laboratory markers of lipid peroxidation, indicating the presence of oxidative stress through the action of RONS on lipids [70].

Our data showed that the Pb group exhibited significantly higher TBARS levels than the Sham group in both brain and lung tissues (*p* < 0.0001; Figure 3), reinforcing the role of RONS as key mediators of oxidative biochemical disturbances induced by experimental malaria.

Previously, studies support our findings. Reis et al. [54] reported increased MDA levels and conjugated dienes in the brains of *P. berghei*‐infected C57BL/6 mice, indicating oxidative stress. Fernandes et al. [71] linked elevated plasma levels of reactive aldehydes in infected mice to malaria severity. Scaccabarozzi et al. [72] showed that *P. berghei* infection in C57BL/6J mice induced biochemical alterations in both liver and lung tissues and contributed to acute respiratory distress syndrome, attributed to an exacerbated oxidative response characterised by increased MDA levels and altered antioxidant enzyme activity. More recently, Chuljerm et al. [73] also reported elevated TBARS levels in the plasma and livers of *P. berghei*‐infected mice.

Our findings indicate that in *P. berghei*‐infected mice, an exacerbated oxidative response appears to predominate, as demonstrated by elevated TBARS levels. This suggests a loss of structural and functional integrity of the cell membrane in brain and lung tissues, which may be closely associated with increased parasitemia.

However, treatment with LYC and NAC significantly reversed the elevation in TBARS levels compared to the Pb group (*p* < 0.0001; Figure 3). Moreover, LYC treatment reduced TBARS levels to values comparable to those observed in the Sham group, suggesting that LYC may be effective in scavenging RONS, inhibiting lipid peroxidation and protecting membrane lipids from oxidative damage in brain and lung tissues during malarial infection.

These data are consistent with previous findings in the literature, which have reported that both LYC and NAC can reduce endogenous oxidant levels and protect cells against a wide range of pro‐oxidative insults by directly reacting with RONS [63, 74, 75]. According to Zhang [76], LYC can inhibit oxidative stress and TBARS production, thereby limiting the generation of RONS. LYC activity has also been implicated in the in vitro elimination of *P. falciparum* [62]. However, its direct effect on parasite proliferation in vivo remains unclear. In contrast, the antioxidant activity of NAC is likely mediated by its free thiol (—SH) group, which acts as an electron donor, facilitating its interaction with RONS.

By promoting cellular damage, parasites can alter the concentration of mobilisable antioxidants in the host, triggering endogenous defence mechanisms against oxidative stress. These defence responses help protect biological membranes, which are particularly susceptible to lipid peroxidation and oxidative injury. Mobilisable antioxidants include enzymatic systems such as SOD, which catalyses the dismutation of O_2_˙^−^ into H_2_O_2_, CAT and GSH‐Px, which reduce H_2_O_2_ and ROOH to less reactive molecules like water, alcohol and oxygen, and non‐enzymatic antioxidants such as GSH [5, 6].

In this context, we observed a significant increase in AC‐ABTS and AC‐DPPH levels in the Pb group compared to the Sham group in both brain and lung tissues (*p* < 0.0001; Figure 4). This elevation in antioxidant capacity likely reflects an adaptive response to increased oxidative burden, indicating that the animals mobilised endogenous antioxidants in an attempt to neutralise the RONS generated during *P. berghei* infection.

Supporting these results, a significant positive correlation was found between TBARS and AC‐ABTS levels (*r* = 0.4; *p* < 0.0001), as well as between TBARS and AC‐DPPH (*r* = 0.5; *p* < 0.0001) in the Pb group. The enhanced antioxidant capacity may be attributed to the upregulation of mobilisable antioxidant enzymes or to the elevated intracellular concentrations of RONS such as H_2_O_2_, O_2_˙^−^, OH˙, ONOO^−^ resulting from the infection.

Conversely, LYC supplementation led to a significant reduction in AC‐ABTS and AC‐DPPH levels compared to the Pb group in both tissues (Figure 4). In the LYC + Pb group, antioxidant capacity was restored to levels comparable to those of the Sham group in both brain and lung tissues. In contrast, NAC treatment led to a significant increase in AC‐ABTS levels in brain tissue compared to the Sham and LYC + Pb groups. In lung tissue, NAC restored AC‐ABTS levels to those observed in the Sham group.

These findings suggest that *P. berghei‐*infected mice, LYC supplementation may reduce the activity of mobilisable antioxidants as part of a cellular redox regulation mechanism triggered by the intake of exogenous antioxidants. In contrast, NAC, as a precursor and analogue of GSH, may promote the replenishment of intracellular GSH levels; the most abundant endogenous antioxidant responsible for protecting cells from oxidative damage.

In this context, previous studies have shown that in diseases where oxidative stress is a key pathogenic mediator, such as Alzheimer's, Parkinson's, Chagas, dengue and malaria, the action of endogenous mobilisable antioxidants alone is often insufficient to maintain redox homeostasis [77, 78, 79, 80].

To counteract the deleterious effects of RONS, supplementation with consumable antioxidants or pharmacological antioxidants, including vitamins E and C, polyphenols (e.g., flavonoids and resveratrol), carotenoids (e.g., β‐carotene and LYC) and agents like NAC is essential for preserving optimal cellular function. LYC, in particular, is a potent antioxidant with additional anti‐inflammatory, anti‐atherogenic, antidiabetic, neuroprotective and anticancer properties [28, 30, 31, 39, 81, 82, 83].

Antioxidants can exert their effects by increasing the concentration of other antioxidants in the body, donating electrons to RONS to neutralise them, or binding directly to these species and inactivating them. Additionally, they may prevent the formation cascade of highly reactive species such as OH˙ and ONOO^−^ [84].

These RONS are considered key triggers of the intense inflammatory response associated with disease progression and poor outcomes in malaria [85]. RONS may also act as second messengers in intracellular signalling pathways, activating mononuclear cells, macrophages and dendritic cells, which subsequently stimulate the release of high mobility group box‐1 (HMGB‐1) into both intra‐ and extracellular compartments [18].

According to Techarang et al. [86], increased HMGB‐1 expression in endothelial cells can activate several receptors, including RAGE, TLR‐4 and TLR‐2. These receptors initiate signalling cascades that activate nuclear factor kappa B (NF‐κB), leading to increased production of pro‐inflammatory cytokines such as TNF‐α, interferon‐gamma (IFN‐γ), IL‐1β and IL‐6, all of which are implicated in the pathogenesis of malaria.

These inflammatory mediators can also stimulate iNOS expression in macrophages. Under normal conditions, iNOS activity in macrophages is low; however, in response to inflammatory signals, such as IFN‐γ, its expression is markedly upregulated, resulting in increased NO production [87].

NO is a multifunctional mediator involved in various physiological processes. It plays a critical role in regulating bronchomotor tone through non‐adrenergic, non‐cholinergic (NANC) neural pathways and serves as an important vasodilator in vascular endothelium tissue [88]. Furthermore, NO is essential for the phagocytic activity of macrophages, contributing to pathogen destruction via the formation of reactive molecules such as ONOO^−^.

Despite its protective role, our results showed that increased NO production was not correlated with a reduction in parasitemia. Instead, we observed a significant increase (*p* < 0.0001) in NO levels in the Pb group compared to the Sham group in both brains and lung tissues throughout the study period (Figure 5). This finding suggests that excessive NO production may have had deleterious effects, such as generalised vasodilation leading to hypotension and oxidative damage caused by ONOO^−^, a highly reactive species capable of inducing lipid peroxidation and membrane damage.

Interestingly, LYC treatment led to a significant reduction in NO level in brain tissue compared to the Pb and NAC + Pb groups (Figure 5), normalising NO concentration to values similar to those of the Sham group. In contrast, in lung tissue, LYC treatment resulted in elevated NO levels in the LYC + Pb group compared to all other groups.

This apparent tissue‐specific divergence may be explained by differential expression of nitric oxide synthase isoforms (iNOS, eNOS, nNOS), as well as variations in local redox environments [89]. In lung tissue, increased NO levels may reflect a beneficial eNOS‐mediated vasodilatory response, which could enhance tissue perfusion and oxygen delivery [59]. Conversely, in brain tissue, reduced NO levels may help prevent ONOO^−^‐induced neurotoxicity.

Moreover, previous studies have shown that LYC treatment can suppress iNOS protein and mRNA expression in lipopolysaccharide‐stimulated RAW 264.7 macrophage cells, suggesting that LYC exerts anti‐inflammatory effects by downregulating iNOS expression at both the transcriptional and translational levels [90]. Although these mechanisms remain speculative in the context of malaria, our findings highlight the complex, tissue‐specific roles of LYC in modulating NO metabolism, warranting further investigation.

Previous studies have demonstrated the protective role of NO in improving cerebral microcirculation and reducing vascular pathology in cerebral malaria [91, 92]. Conversely, in malarial infections, ONOO^−^ can induce protein nitration, depletion of consumable antioxidants and induce peroxidation of the microvascular endothelium [22]. These effects contribute to a marked reduction in blood flow, leading to ischemia, hypoxia, vasospasms and tissue hypoperfusion, as well as cell‐mediated congestion that may culminate in pulmonary edema and stroke [93, 94]. Additionally, tissue ischemia may be exacerbated by cytoadhesion of parasitized erythrocytes, a hallmark of *Plasmodium* infection [5, 12].

Prolonged hypoxia disrupts mitochondrial oxidative phosphorylation, resulting in ATP degradation and accumulation of xanthine oxidase and hypoxanthine. In an attempt to restore tissue oxygenation, the body stimulates eNOS expression to elevate NO production and promote reperfusion. However, upon reperfusion, xanthine oxidase catalyses the oxidation of hypoxanthine, producing O_2_˙^−^, H_2_O_2_ and uric acid, the latter of which was used in the present study as a marker of IRS. In such contexts, O_2_˙^−^ may further react with NO to generate ONOO^−^, exacerbating oxidative damage [95].

In this context, our data indicated that *P. berghei* infection significantly increased UA levels in the Pb group compared to the Sham group in both tissues (*p* < 0.0001; Figure 6). In contrast, LYC supplementation significantly reduced UA levels in both tissues in relation to the Pb group (*p* < 0.0001), and also in comparison to the NAC + Pb group in brain tissue. Moreover, LYC treatment normalised UA levels in both tissues. A similar normalisation was observed in lung tissue following NAC treatment, suggesting a protective role for both compounds against damage caused by IRS.

These findings align with previous studies reporting elevated UA levels in children infected with *P. falciparum* during acute episodes, with correlations to disease severity, highlighting UA as an important mediator in the pathophysiology of malaria [96].

Given its favourable safety profile and natural dietary origin, LYC represents a promising candidate for adjuvant therapy in malaria. Although the present findings are limited to a murine model, they offer foundational evidence to support the translational potential of LYC supplementation in clinical settings. Future studies should explore its combined efficacy with standard antimalarial therapies, determine optimal dosing regimens in humans, and assess its bioavailability during infection. Furthermore, considering the broad availability of LYC‐rich foods, nutritional strategies incorporating such sources may offer a complementary approach to support malaria prevention and management in endemic regions.

## 5 Conclusions

LYC prevented oxidative damage induced by *P. berghei* infection in the brain and lung tissue of mice by restoring levels of NO, TBARS and antioxidant molecules. Additionally, LYC supplementation mitigated the onset of ischemia and reperfusion syndrome, commonly associated with malaria pathology. These findings provide compelling evidence for the protective role of LYC against oxidative stress in an in vivo experimental model of malaria and underscore the potential of antioxidant therapy as an adjunctive strategy in malaria management. Therefore, LYC administration emerges as a viable, safe and innovative therapeutic approach for reducing the oxidative damage associated with malarial infection, warranting further investigation in clinical settings.

## Author Contributions

Conceptualization: E.L.P.V., M.I., S.P. Methodology, E.L.P.V., A.R.Q.G., A.d.S.B.d.S., M.d.S.G., E.P.d.C. Formal analysis: E.L.P.V. Investigation, E.L.P.V. Data curation: E.L.P.V. and S.P. Writing – original draft preparation. E.L.P.V., A.R.Q.G., A.d.S.B.S., M.d.S.G., E.P.d.C., O.O.F., M.S.d.O. Writing – review and editing: E.L.P.V., E.H.d.A.A., M.I., S.P. Visualisation: S.P. Supervision: S.P., M.I. Project administration: S.P. Funding acquisition: S.P. All authors have read and agreed to the published version of the manuscript.

## Ethics Statement

The animal study protocol was approved by Comissão de Ética no Uso de Animais da UFPA (CEUA/UFPA; Protocol Code No. 3235130919, issued on September 10th, 2021).

## Conflicts of Interest

The authors declare no conflicts of interest.

## Data Availability

The data that support the findings of this study are available on request from the corresponding author. The data are not publicly available due to privacy or ethical restrictions.
